# Association between open-angle glaucoma and Alzheimer’s disease in Sweden: a long-term population-based follow-up study

**DOI:** 10.48101/ujms.v126.7819

**Published:** 2021-06-21

**Authors:** Curt Ekström, Ida Puhto, Lena Kilander

**Affiliations:** a Ophthalmology, Department of Neuroscience, Uppsala University, Uppsala, Sweden; b Geriatrics, Department of Public Health and Caring Sciences, Uppsala University, Uppsala, Sweden

**Keywords:** Alzheimer’s disease, open-angle glaucoma, normal-tension glaucoma, dementia, epidemiology, risk factor

## Abstract

**Background:**

Open-angle glaucoma (OAG) and Alzheimer’s disease (AD) are two age-related neurodegenerative diseases of significant public health importance. Epidemiological studies have indicated that there might be an association between the disorders.

**Methods:**

Predictors of AD, including mixed and unspecified dementia, were analysed in a cohort of 712 residents aged 65–74 years, examined in a population survey in the rural district of Tierp, Sweden, from 1984 to 1986. To expand the sample size, 821 people were recruited by means of glaucoma case records established at the Eye Department in Tierp from 1978 to 2007. In this way, the cohort comprised 1,533 people, representing more than 21,000 person-years at risk. Medical records were reviewed to identify subjects diagnosed with dementia. Those with a follow-up duration shorter than 2 years were excluded.

**Results:**

By the conclusion of the study, in August 2020, 307 subjects had received a diagnosis of AD, including mixed and unspecified dementia. Of these cases, 55 were affected with definite OAG at baseline. Higher age and ischemic heart disease were the only predictors of AD identified. In multivariate analysis, adjusting for age, participation in the population survey and competing events, no association was found between OAG and AD (hazard ratio 1.08; 95% confidence interval: 0.80–1.47).

**Conclusion:**

In this long-term follow-up study of subjects aged 65–74 years old in Sweden, OAG was not associated with AD.

## Introduction

Open-angle glaucoma (OAG) and Alzheimer’s disease (AD) are two progressive, age-related neurodegenerative diseases of significant public health importance. Glaucoma is characterised by loss of optic nerve fibres with typical appearance of the optic nerve head and consistent visual field defects, whilst AD is known for its ongoing cognitive decline and behavioural impairment. Neuropathologically, hallmarks of AD include the accumulation of large extracellular β-amyloid plaques and intracellular fibrillar tangles of abnormally phosphorylated tau protein within the central nervous system (1). Polymorphism of the apolipoprotein E (APOE) gene, involved in the transport of lipids, is an important risk factor for late onset AD (2). At the same time, increased intraocular pressure (IOP) is closely related to OAG (3).

Similarities between OAG and AD have raised the question of whether they share common risk factors or if one condition has an influence on the other. Two main hypotheses have been proposed to explain a possible connection between the disorders. The first hypothesis includes a common neurodegenerative process involving the activation of enzymatic caspases and the production of neurotoxic amyloid β (4). The findings of optic nerve degeneration and the loss of retinal ganglion cells in AD (5, 6) agree with this assumption. The second hypothesis implicates optic nerve damage as a consequence of an abnormally high-pressure difference across the lamina cribrosa in AD, with decreased cerebrospinal fluid pressure (7). Berdahl et al.’s study supports this concept (8). They found lower cerebrospinal fluid pressure in patients with OAG than in controls.

Numerous researchers have examined a possible association between OAG and AD, albeit with conflicting results. In a case–control study in Bavaria, Bayer et al. found an increased rate of glaucoma in AD patients (9). Likewise, Tamura et al. reported a high frequency of OAG in Japanese patients with AD (10). Interestingly, they also found a connection between APOE genotypes and OAG. In a 3-year follow-up study, Helmer et al. revealed a relationship between OAG and incident dementia (11). Moreover, two register-based studies in Taiwan found a 40 and 47% increased risk, respectively of AD, in subjects with a diagnosis of OAG (12, 13). Another Taiwanese study demonstrated a 52% increased risk in normal-tension glaucoma (NTG) compared with subjects without glaucoma (14). Finally, a case–control study recently reported that cognitive impairment was more prevalent in OAG subjects with an IOP ≤21 mmHg than in those with an IOP ≥25 mmHg (15).

In contrast, several large-scaled cohort studies have failed to confirm the results (16–19). Furthermore, in a cross-sectional study from Singapore, glaucoma was not associated with cognitive dysfunction (20). Clearly, additional research is essential for better understanding of the relationship between these common diseases.

The purpose of the present research was to explore the relationship of OAG with AD, including mixed and unspecified dementia, in a Swedish cohort. The investigation took the form of a long-term follow-up study on a defined population. Study results have previously been reported briefly (21).

## Methods

### The study population

Residents in two rural districts in Uppsala county, south-central Sweden, registered with a glaucoma case record at the Eye Department in Tierp, or who had participated in the Tierp Glaucoma Survey, were eligible for the study. The inclusion criteria embraced all subjects 65–74 years of age.

### The Tierp Glaucoma Survey

In 1984–1986, a population survey was conducted in the rural district of Tierp. Its target population comprised 2,429 residents, aged 65–74 years old. A representative sample of about one-third of the target population was randomly selected. Of the eligible number of 838 residents, 760 (91%) underwent a detailed eye examination, as described elsewhere (22). The study was primarily designed to address the distribution and determinants of OAG. However, a vast amount of information was collected, including data on health problems. Briefly, an interview was first held, covering medical and family history. Information was also obtained from medical records. Visual fields were tested using the Competer 350 automated perimeter (Bara Elektronik AB, Lund, Sweden). After perimetry, the pupils were dilated, and the slit lamp biomicroscopy, including a binocular assessment of the optic discs and gonioscopy, was undertaken. Pseudoexfoliation was defined as the presence of characteristic white flakes on the lens capsule or on the pupillary border.

### The cohort

A total of 78 residents did not participate in the population survey. Of these, one joined the cohort after being examined in 1993. Thus, this part of the cohort was comprised of 761 subjects. To expand the sample size, 923 patients were recruited by means of glaucoma case records established in 1978–2007. Those enrolled had a diagnosis of ocular hypertension, glaucoma, suspicious optic discs or a positive family history. In addition, more than 200 subjects had participated as controls in a case–control study on risk factors for OAG (unpublished data). Apart from visual field testing, they underwent an eye examination equal to that of the population survey. Information about the visual fields was obtained from medical records. The examination day was defined as the index date for the participant in the population survey. For the rest of the cohort, the first visit at the Eye Department at the age of 65–74 years was chosen as the index date.

**Figure 1:**
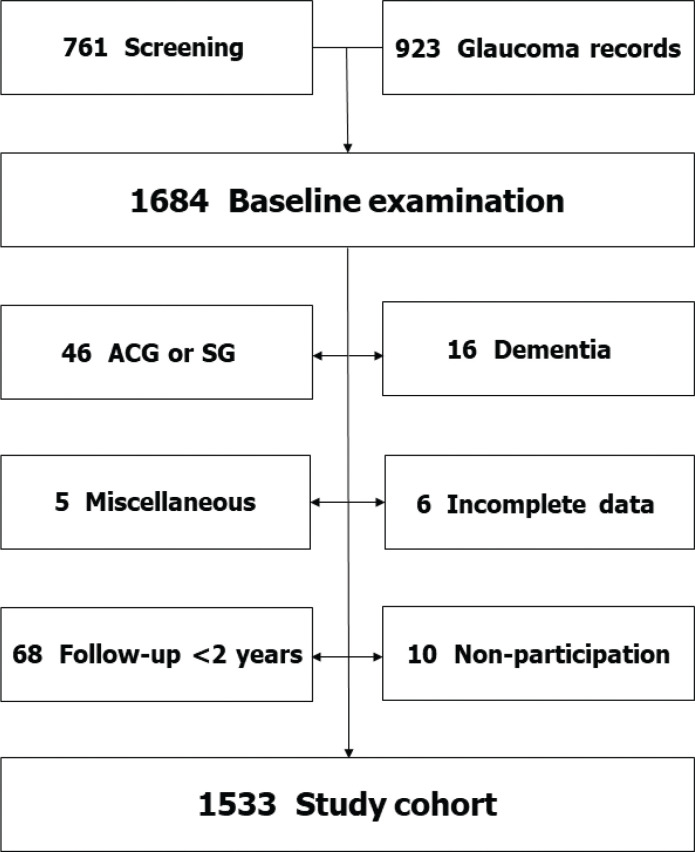
Flow chart showing how the study cohort of 1,533 individuals was derived. ACG, angle-closure glaucoma; SG, secondary glaucoma. The miscellaneous group included five subjects not examined in Tierp.

Of the total number of 1,684 individuals, 46 were diagnosed with either angle-closure glaucoma or secondary glaucoma. These individuals were excluded from the study, as they were 16 subjects with dementia or mental retardation, six with incomplete data and five for other reasons. Only subjects who completed a follow-up time of at least 2 years (‘immortal person–time’) were accepted. Consequently, 68 subjects were removed from the study. Ten people did not want to participate (Figure 1). The remaining 1,533 constituted the study cohort, whose characteristics are presented in Table 1. The Regional Ethical Review Board of Uppsala University approved the study. The tenets of the Declaration of Helsinki were observed.

**Table 1:** Characteristics of the cohort, by age and gender.

Age group	No. of people (*n* = 1,533)		Person–years (*n* = 21,676)	
	Female (%)	Male (%)	Female (%)	Male (%)
65-69 years	416 (48)	312 (47)	6,978 (53)	4,588 (54)
70-74 years	455 (52)	350 (53)	6,194 (47)	3,916 (46)
65-74 years	871 (100)	662 (100)	13,172 (100)	8,504 (100)

Mean follow-up time: 14.1 years (standard deviation: 7.0 years).

### Assessment of dementia

Follow-up started after the baseline examination and ended on 31 August 2020. Medical records were reviewed to identify subjects diagnosed with dementia. If the word dementia was found anywhere in the text, pertinent parts of the records were copied and de-identified. Permanent impairment in cognitive and social function, persisting for at least 6 months, had to be present for a diagnosis of dementia. A geriatrician (LK), who was not cognisant of the baseline data, approved the diagnoses using all available information. Diagnoses of AD fulfilled the National Institute on Aging and the Alzheimer’s Association criteria (23). Thus, a history free from abrupt onset and computed tomography without sign of any major cerebrovascular disease was mandatory for a diagnosis of ‘pure AD’. Cases with insidious onset and having a slowly progressive course, showing evidence of cerebrovascular lesions according to tomography, were identified as having mixed AD and vascular dementia. Most patients with an onset after 80 years of age were designated as having unspecified dementia. For this study, ‘pure AD’ was combined with mixed and unspecified dementia into a single category.

### Classification of open-angle glaucoma

Consistent with the concept of Foster et al. (24), glaucoma with pseudoexfoliation was classified as OAG. NTG was defined as a variant of OAG in cases where not more than one IOP reading exceeding 21 mmHg had ever been recorded and none of the readings were above 24 mmHg. To qualify for a diagnosis of OAG, a reproducible visual field defect was a prerequisite, consistent with glaucoma and not explicable on other grounds. Likewise, subjects with end-stage disease in either eye were included among the OAG cases. In all, 264 subjects fulfilled a diagnosis of definite OAG, 42 in the population sample and 222 in the rest of the cohort. Pseudoexfoliation in either eye was present in 152 (57.6%) of the subjects with OAG.

### Statistical methods

Age- and sex-standardised morbidity ratios (SMRs) were estimated. Follow-up time was calculated from the index date to the date of the dementia diagnosis (*n* = 357), death (*n* = 1,049), migration out of Uppsala county (*n* = 46) or the end of the study (*n* = 81), whichever occurred first. ‘Immortal person–time’ was removed from the follow-up time for all calculations (25).

Following standardised analyses, Cox proportional hazards models were developed to assess the effect of more than one predictor on the risk of AD, censoring those who died, migrated from the county, received other types of dementia diagnosis or remained in the cohort at the end of the follow-up period. Adjustments were made for the influence of censuring due to death (competing events). Only predictors with a substantial number of exposed cases were used in the multivariate analyses. The proportional hazard assumptions were tested using time-dependant covariates. The effect of the covariates on survival was independent on time, apart from participation in the population survey. Consequently, a time-dependant variable was included in the Cox models.

## Results

The median follow-up time for survivors was 18.9 years (range 13.6–34.8 years). By the end of the study, 357 cases of dementia had been identified, 307 of whom had ‘pure AD’, mixed dementia or unspecified dementia. Of the 307 cases, 55 had definite OAG in either eye at baseline. ‘Pure AD’ or mixed dementia was found in 50 subjects, whilst 257 were diagnosed with unspecified dementia.

Higher age was the only variable found to be associated with AD. Subjects aged ≥70 years experienced a 1.69-fold (95% confidence interval [CI]: 1.35–2.13) increased risk, compared with those <70 years (Table 2). Adjusting for age, the risk of developing ‘pure AD’ or mixed dementia in OAG patients was lower than the risk for any type of dementia, SMR 0.61 (95% CI: 0.24–1.54) and 1.12 (95% CI: 0.85–1.48), respectively. There was no association between participation in the population survey and AD (SMR 0.83; 95% CI: 0.66–1.05). NTG was present in 11 subjects, one of whom was diagnosed with unspecified dementia.

**Table 2:** Associations of potential risk factors and Alzheimer’s disease including mixed and unspecified dementia in a cohort of 1,533 individuals, adjusted for age and sex.

Baseline characteristics	No. of cases	SMR (95% CI)
	(*n* = 307)	
**Age ≥ 70 years^a^**		
No	126	1.00
Yes	181	1.69 (1.35–2.13)
**Female sex^b^**		
No	103	1.00
Yes	204	1.23 (0.97–1.55)
**Participation in the population survey**		
No	180	1.00
Yes	127	0.83 (0.66–1.05)
**Open-angle glaucoma, either eye**		
No	252	1.00
Yes	55	1.16 (0.86–1.56)
**Pseudoexfoliation, either eye**		
No	215	1.00
Yes	92	1.05 (0.82-1.34)
**Smoking status**		
Never smoked	224	1.00
Past smoker	57	0.97 (0.70–1.35)
Current smoker	26	0.84 (0.55–1.30)
**Diabetes mellitus**		
No	276	1.00
Yes	31	1.02 (0.70–1.48)
**Hypertension, treated**		
No	196	1.00
Yes	111	1.15 (0.91–1.45)
**Ischaemic heart disease**		
No	256	1.00
Yes	51	1.27 (0.94–1.72)

CI: confidence interval; SMR: standardised morbidity ratio.

a Adjusted for sex.

b Adjusted for age.

Cox proportional hazards models included OAG, age, sex, participation in the population survey, smoking habits, systemic hypertension and ischaemic heart disease (Table 3). No association was found between OAG and AD (hazard ratio [HR] 1.08; 95% CI: 0.80–1.47). Every year of higher age increased the risk by 16% (HR 1.16; 95% CI: 1.12–1.21). In a separate model, ischemic heart disease was found to be associated with AD, including mixed and unspecified dementia (HR 1.43; 95% CI: 1.05–1.94). Inclusion of birth year in the models had no effect on the estimates (data not shown).

**Table 3:** Association of open-angle glaucoma with Alzheimer’s disease including mixed and unspecified dementia in a cohort of 1,533 individuals.

OAG	No. of subjects	No. of cases	Hazard ratio (95% confidence interval)	
			Age-adjusted^a^	Multivariate^b^
No	1,269	252	1.00	1.00
Yes	264	55	1.14 (0.85–1.53)	1.08 (0.80–1.47)

OAG: open-angle glaucoma.

a Adjusted for age (continuous variable) and competing events (deaths).

b Adjusted for age (continuous variable), participation in the population survey, including a time-dependent variable, and competing events (deaths).

## Discussion

To our knowledge, this study on a defined population is the longest follow-up study on OAG and the risk of developing AD yet to be reported. Definite OAG was found to be unrelated to AD. However, although we had access to a sizable cohort, only 55 AD cases had been exposed to definite OAG, limiting the ability to reveal a small increase in risk and to analyse sub-groups. In fact, applying a confidence level of 95% and a power of 80%, this study had the capacity to detect a 53% increased risk.

Contrary to the present study, a meta-analysis of six studies on OAG and AD by Xu et al. recently showed a 17% increased risk of AD (26). There are several explanations for the discrepancy between our study and other studies. Clearly, insufficient statistical power downgrades the chance of detecting an enlarged risk. Moreover, the study in Tierp was a long-term follow-up study, whilst most of the studies demonstrating a connection with AD referred to previously were either case–control studies or register-based studies (9–11, 13–15). It is well known that case–control studies are more susceptible to bias than cohort studies. Register-based studies, on the other hand, are reliant on the quality of the registers used. Of interest, in the review by Xu et al., there was no association between OAG and AD when the analysis was restricted to cohort studies (26).

Furthermore, the studies showing a relationship between OAG and AD, where information on the IOP was provided, were generally characterised by an IOP within the normal range (10–12). In contrast, increased IOP was a typical finding of OAG diagnosed in the Tierp population (27). In the present study, 11 out of 264 patients with OAG were found to have NTG. Of note, two studies recently have reported a relationship of NTG with AD (14, 15), though a previous Danish study did not (28). There are evidence suggesting that NTG and AD share common risk factors. A cross-sectional study from South Korea found a higher risk for vascular and metabolic comorbidities in subjects with OAG and a baseline IOP ≤15 mmHg (29). Moreover, a Swedish cohort study confirmed a relationship between vascular risk factors and dementia (30). In fact, if NTG is related to AD, it is possible that some of the divergence between the result in Tierp and other studies might be explained by variances in the characteristics of OAG cases.

Results from the study in Tierp have previously been reported briefly (21). Although the follow-up time for surviving individuals increased by 4 years in the present study and an additional 31 cases of dementia were identified, the risk associated with OAG was almost identical, demonstrating stability in the data over time. Also, the increased number of cases facilitated a sensitivity analysis, showing inconsistency with risk estimates. Thus, the risk of ‘pure AD’ or mixed dementia in OAG was substantially lower than the risk of any type of dementia (SMR 0.61 and 1.12, respectively). However, considering the low number of ‘pure AD’ or mixed cases, the finding should be interpreted with caution.

The study in Tierp involved people aged 65–74 years old, whilst the other studies referred to in this report covered a broader age span. Nonetheless, there are no reports of age differences in AD risk associated with OAG.

Our study has several strengths, including its community-based design, long-term follow-up with risk factors measured at baseline before disease diagnosis and sizeable cohort, nearly half of which was randomly selected. Furthermore, the same glaucoma specialist conducted all the eye examinations. A geriatrician (LK), ‘masked’ to baseline information, approved the dementia diagnoses. Moreover, a reproducible visual field defect or end-stage disease was required for a diagnosis of OAG. Nevertheless, as with many epidemiologic investigations, the research is limited in several respects.

First, baseline examination dates extended over nearly 30 years, which might give rise to bias. However, adjustment for year of birth in the regression models did not change the estimates. Second, some misclassification of OAG diagnoses cannot be ruled out. If OAG increases the risk of AD, this type of information bias should be non-differential, thereby ‘diluting’ the effect of OAG in the analyses. On the other hand, if OAG has no effect, non-differential misclassification would not bias the result (31). Third, dementia diagnoses were usually based on clinical judgement by general practitioners, after which an experienced geriatrician (LK) classified the cases by reviewing the medical records. The extent to which doctors make a diagnosis of dementia in cognitive impaired patients varies. Consequently, an unknown number of people with dementia were likely to have remained undiagnosed or were not diagnosed until they had reached the stage of advanced disease. There is no reason to believe that decisions made by general practitioners were in any way connected with the exposure being studied, and therefore had any effect on risk estimates.

Finally, ‘pure AD’ or mixed dementia was diagnosed in 14% of the cases, which is much lower than expected (32). A probable explanation for the paucity of AD cases is that specialists in geriatrics were involved in only a small proportion of cases. Moreover, strict diagnostic criteria were applied to establish a diagnosis of AD for this study. We believe that the distribution of dementia diagnoses justifies our decision to combine AD, mixed dementia and unspecified dementia into a single category, as many patients classified with unspecified dementia most likely had AD. The proportion of AD, mixed AD and vasculardementia, as well as unspecified dementia, stands at approximately 70% in the National Swedish Dementia Registry (33).

In conclusion, in this long-term follow-up study of subjects aged 65–74 years old in Sweden, definite OAG was not related to AD. Thus, we were unable to confirm the association found in some previous studies.
